# Bridging Educational Gaps in Mid- to Late-Life Cognitive Function Through Active Cognitive Engagement

**DOI:** 10.1093/geroni/igaf122.3551

**Published:** 2025-12-31

**Authors:** Thakshila Dasanayake, Nikki Hill, Diane Berish, Jacqueline Mogle

**Affiliations:** The Pennsylvania State University, University Park, Pennsylvania, United States; The Pennsylvania State University, University Park, Pennsylvania, United States; The Pennsylvania State University, University Park, Pennsylvania, United States; RTI Health Solutions, Durham, North Carolina, United States

## Abstract

Higher education levels may establish a foundation for ongoing intellectual stimulation throughout life, leading to better cognition, including episodic memory (EM) and executive functioning (EF). Additionally, cognitive activity engagement may mitigate declines in cognition as individuals age. However, the relationship between the potential compensatory benefits of education and late-life cognitive engagement is somewhat unclear. This study examined the interaction between level of education and current frequency of cognitive activity on EM and EF in adulthood, using participants from the second wave (2004–2006) of the Midlife in the United States study (n = 3,462; Mage = 55.99, SD = 12.21; range: 32–84 years). Principal component analysis was used to calculate composite cognitive activity scores (intellectual, problem-solving, technological, and social domains) from self-reported frequencies of engagement (never to daily). Multiple linear regressions, controlling for covariates, tested associations of education, cognitive activity, and their interaction, followed by age-stratified analyses (32–64, 65–74, and 75+ years). Significant negative interactions between education and cognitive activity were observed for both EM (β = –0.010, p = .042) and EF (β = –0.009, p = .030), indicating that the cognitive advantage of higher education is smaller at higher cognitive activity levels. Both education and cognitive activity independently predicted better EM and EF. The compensatory effect was stable across age groups for EM, whereas education’s benefit for EF was smaller in older adults (p = .037), though not moderated by activity. Findings support active cognitive engagement as a strategy to reduce education-related disadvantages in cognition.

